# Minding the Gatekeepers: Referral and Recruitment of Postpartum Mothers with Depression into a Randomized Controlled Trial of a Mobile Internet Parenting Intervention to Improve Mood and Optimize Infant Social Communication Outcomes

**DOI:** 10.3390/ijerph17238978

**Published:** 2020-12-02

**Authors:** Kathleen M. Baggett, Betsy Davis, Lisa B. Sheeber, Robert T. Ammerman, Elizabeth A. Mosley, Katy Miller, Edward G. Feil

**Affiliations:** 1 Mark Chaffin Center for Healthy Development, Georgia State University, Atlanta, GA 30303, USA; emosley@gsu.edu (E.A.M.); kspinks@gsu.edu (K.M.); 2 Oregon Research Institute, Eugene, OR 97403, USA; betsy@ori.org (B.D.); lsheeber@ori.org (L.B.S.); edf@ori.org (E.G.F.); 3 Cincinnati Children’s Hospital Medical Center, University of Cincinnati College of Medicine, Cincinnati, OH 45229, USA; robert.ammerman@cchmc.org

**Keywords:** maternal depression, referral, recruitment, mobile intervention, clinical trials

## Abstract

Mothers in the United States (U.S.) who are of non-dominant culture and socioeconomically disadvantaged experience depression during postpartum at a rate 3 to 4 times higher than mothers in the general population, but these mothers are least likely to receive services for improving mood. Little research has focused on recruiting these mothers into clinical intervention trials. The purpose of this article is to report on a study that provided a unique context within which to view the differential success of three referral approaches (i.e., community agency staff referral, research staff referral, and maternal self-referral). It also enabled a preliminary examination of whether the different strategies yielded samples that differed with regard to risk factors for adverse maternal and child outcomes. The examination took place within a clinical trial of a mobile intervention for improving maternal mood and increasing parent practices that promote infant social communication development. The sample was recruited within the urban core of a large southern city in the U.S. and was comprised primarily of mothers of non-dominant culture, who were experiencing severe socioeconomic disadvantage. Results showed that mothers self-referred at more than 3.5 times the rate that they were referred by either community agency staff or research staff. Moreover, compared to women referred by research staff, women who self-referred and those who were referred by community gatekeepers were as likely to eventually consent to study participation and initiate the intervention. Results are discussed with regard to implications for optimizing referral into clinical intervention trials.

## 1. Introduction

Ramifications of depressive conditions are quite severe, with depression being a leading cause of disability for women and contributing significantly to the overall burden of disease globally [1]. During the first year after childbirth, women are more likely to develop depression and anxiety than at any other time in their life [2]. Perhaps some of the greatest costs of maternal depression are borne by the children. Perinatal mood and anxiety disorders compromise parenting and adversely affect children’s physical and emotional development [3,4,5]. In particular, maternal depression can undermine sensitive and responsive caregiving, parenting behaviors that are key to supporting healthy child development [6]. Hence, it is crucial to provide interventions that both address maternal depression and strengthen skills involved in sensitive and responsive parenting as early as possible in a child’s life to promote subsequent maternal and child health and development [7].

Unfortunately, delivering intervention to mothers and children most in need has proven difficult, and few depressed mothers receive treatment [8]. Mothers living in socioeconomic disadvantage and those who are of non-dominant culture are more likely to experience depression compared to higher resourced mothers of the dominant culture [9], but they are far less likely to receive treatment [10]. In the U.S., women of European origin use mental health services at more than twice the rate of Black or Latinx women [11]. This finding is consistent with the more general finding that racial and ethnic minorities are less likely to receive mental health services when compared to non-Latinx White persons after controlling for multiple demographic characteristics and disorder severity [12]. The American Academy of Pediatrics (AAP) and the American College of Obstetricians and Gynecologists (ACOG) [13] have called for women to receive depression screening and referral to intervention during the first year postpartum [14]. Evidence suggests, however, that the majority of women with depressive symptoms do not receive screening and appropriate treatment [15], even within systems reportedly conducting universal screening [16].

According to the Maternal, Infant, and Early Childhood Home Visiting Technical Assistance Coordinating Center [17], mothers who are least likely to enroll and engage in services are those experiencing depression and, because of structural and systemic biases [18], are of non-dominant culture and living within socioeconomic disadvantage. Moreover, in the U.S., redistribution of social safety net resources away from the very poor affect their ability to receive needed mental health and other family services [19]. Issues of diversity and access also come into play in research examining efficacy and effectiveness of interventions [20]. Systemic and structural barriers to recruitment into clinical trials of potential participants from non-dominant cultures exist, including distrust in research as well as costs and logistics that impede participation [21]. Moreover, even when members of non-dominant ethnic and racial groups are included in samples, frequent failure to report ethnic and racial characteristics restricts understanding about service delivery to these populations [22]. In a review of clinical trials for depression spanning a 36-year period [23], researchers reported that over time, participation by persons of low socioeconomic status (SES) and those of minority ethnic and racial backgrounds has increased. Nonetheless, persons of European backgrounds remained the most highly represented group. Moreover, within National Institutes of Health (NIH)-funded clinical trials where racial/ethnic representation in samples is expected and reported [24], researchers often do not examine racial/ethnic status as moderators of treatment effects, such that limited data are available that speak to effectiveness of intervention within these populations [25]. These limitations in the literature leave in question whether access to effective interventions is equitable across populations.

Recruitment of depressed participants, in general, into clinical trials is difficult, spurring conceptual work seeking to understand important gatekeeper-patient factors in recruitment [26] as well as intervention work seeking to modify recruitment behaviors within gatekeeper systems [27]. However, lower resourced individuals are not well-represented within these efforts. One study that directly assessed family general practitioner gatekeeper referrals found substantial disparities in referral and access to mental health services both within and outside of family general practitioner gatekeeper systems [28]. Individuals with greater resource levels were more likely to bypass the gatekeeper system to access directly mental health services, and if these individuals first went to the gatekeeper for referral, they were more likely and more quickly referred to mental health services compared to individuals with lower resource levels [28]. However, this study occurred outside of the U.S. in a country with a profoundly different health service context. In the U.S., large numbers of individuals from non-dominant cultures are under- or uninsured, contributing barriers to health-related service receipt [29] as well as inclusion in clinical trials [30].

Given the ubiquity of digital technologies, mobile health interventions can serve as a way to increase intervention access for those who are traditionally missed [31]. However, there are issues related to what we know about connecting individuals to these interventions. While online recruitment is frequently used in mobile health interventions, very little systematic research exists on this recruitment method [32]. One study comparing recruitment approaches found online paid advertising was more cost effective and timely than provider referral for recruitment to a mobile health early parenting obesity prevention intervention [33]. Another study compared non-paid and paid recruitment approaches to a mobile health smoking cessation intervention and found that online paid advertising and survey panel approaches were best for increasing racial/ethnic diversity in their sample to reach at minimum 25% [34]. Their resultant sample, however, was still primarily White. A recent study describing the recruitment of depressed individuals into a multi-site trial noted that Black participants self-referred into their study at 1.5 times the rate of the local population [35]. However, while self-referral appears an important mechanism for increasing access, the study pertained to a community-based group intervention rather than a mobile health intervention.

At present, existing recruitment studies are extremely limited with regard to implications for referral and recruitment into mobile health intervention trials among non-dominant culture mothers who are depressed and experiencing significant socioeconomic disadvantage. Efforts are needed to identify referral methods that best connect these mothers with intervention and to examine the extent to which various methods are successful at engaging mothers in intervention. We are currently conducting a clinical trial evaluating the efficacy of an integrated internet-based parenting and depression intervention. The intervention is designed to reduce maternal symptomatology and increase sensitive and responsive parenting in mothers of infants. This study, which takes place within the urban core of a large southern city within the U.S., provides a valuable in situ context within which to examine referral and recruitment efforts. Results have the potential to provide information with relevance to improving access to interventions for both depressed women and for women who are socioeconomically disadvantaged, of culturally non-dominant groups, and who continue to have severely unequal access to needed services.

This current study compared three referral approaches to examine their relative success at engaging potential participants in the study. Because researchers may succeed at or fail at engaging women who could benefit from an intervention at multiple points in the recruitment process, we compared the relative success of the referral approaches at several points between initial referral and engagement in the intervention, as described more specifically below. The current study used data from the ongoing clinical trial to examine a year-long period of referral to and recruitment into the intervention trial. The trial provides a unique context within which to compare the success of three referral approaches and examine, in a preliminary way, whether samples referred to the study by different approaches differ on variables associated with adverse maternal and infant outcomes as well as ability to access treatment. The questions we address are:

(1) Are the three referral approaches (i.e., community agency referral, research-team referral, and maternal self-referral) differentially successful as defined by: (a) number of mothers referred; (b) number who complete eligibility screening; (c) number of screenings resulting in eligible participants; (d) number of mothers who consent to participation; and ultimately, (e) number of mothers who initiate intervention?
(2) Do samples referred to the study by different approaches differ on variables associated with maternal or infant outcomes and ability to access treatment, including educational level, relationship status, maternal knowledge of infant development, and severity of maternal symptomatology. Notably, we could not examine income or racial/ethnic differences between groups because of homogeneity in the sample.

## 2. Materials and Methods

The design of the intervention trial, from which the data presented here were derived, called for recruitment of depressed mothers of infants under one year of age. We focused our recruitment efforts to generate a sample inclusive of mothers from non-dominant cultures who were experiencing socioeconomic disadvantage and elevated maternal depressive symptoms. Inclusion criteria were intended to produce a sample of mother–infant dyads, in which infants were at elevated risk for poor social communication development as a function of maternal depression and adverse mother–infant interactions that exacerbate the detrimental effects of poverty. Prior to initiating human subject activity, all study procedures were approved by the Georgia State University IRB. Potentially eligible women were contacted by research staff who described the project, conducted eligibility screening, and obtained informed consent.

Consented participants were randomized into one of two parallel intervention arms: (1) Mom and Baby Net (MBN) or (2) Depression and Developmental Awareness (DDAS). MBN is a 14-session, coach-facilitated, online intervention that teaches mothers both cognitive-behavioral strategies to reduce depressive symptoms and specific skills for engaging with their infants to promote infant social-communication competencies. DDAS is an informational program designed to improve maternal awareness of depression and understanding of infant developmental milestones. The MBN is a skill-based program designed to promote parental competencies to address affective symptoms and interact positively with their infants. DDAS, on the other hand, is an informational program that provides relevant content but does not shape new skills directly. The two mobile interventions were identical with regard to number of sessions, session length, and delivery mechanisms. For more information about the interventions, see Baggett el al. [36].

In this report, we compared three recruitment strategies for enrolling women into the intervention study. The enrollment period examined herein began one year after initiating outreach to build recruitment capacity and continued for an additional 12 months. Recruitment strategies included the following: (1) community agency referrals; (2) research staff outreach visits to community agencies and community events (i.e., research staff referrals); and (3) maternal self-referral. Outcomes compared across the referral conditions included the following: number referred, number screened, number of eligible screens, number of consented, and number that completed initial session to connect with intervention. We also examined a number of individual characteristics that present risk for maternal depression, adverse mother–infant interactions, and poor infant social communication development. These factors included education level, absence of social support, depression symptom severity, and knowledge about infant social communication development.

### 2.1. Sample

Participants referred were mothers of infants aged 0–12 months (*N* = 203). Mothers were included in the study sample if they had a score of 3 or more on the Patient Health Questionnaire-2 (PHQ-2) [37] at screening, were a minimum of 18 years old, spoke English, and lived in the local metropolitan area of a large southern city in the U.S. Exclusion criteria included history of psychotic symptoms, residence in homeless or domestic violence shelter, infant receiving intensive medical treatment, and not having permanent legal guardianship of infant. Demographic characteristics for the sample of 86 enrolled mothers are presented in Table 1.

### 2.2. Referral

At the onset of study referral, we anticipated enrolling approximately 50 women per year based on referral agreements secured from agencies serving socioeconomically disadvantaged mothers and their infants. However, after one year of community outreach and recruitment capacity building, we had obtained only 5 referrals from community agencies. Moreover, we had received 10 maternal self-referrals, and 19 referrals of women our research team recruited at community events and occasional visits to community agencies to provide support to agency staff making referrals. At this point, we broadened our recruitment strategy to include online maternal self-referral. Referral strategies are described below.

Agency referral: Consistent with the original research plan, the research team encouraged ongoing referral from community agencies serving mothers and infants, such as WIC (Special Supplemental Nutrition Program for Women, Infants, and Children), the regional children’s hospital, and medical clinics serving low income women. Partnerships with these agencies were established prior to study initiation for the purpose of participant recruitment. This approach had the potential benefit of reaching women at convenient access points, where they intersected with community agencies designed to promote the health and safety of their children as well as their own well-being. Agencies were provided with information about the project and agreed to screen women with the PHQ-2 and refer potential participants to the project via any of the following mechanisms, as per agency staff preference: (1) use of the project’s online screening and referral system; (2) phone; or (3) secure email. However, many agency staff referred mothers without conducting depression screening; in these cases, the research team completed depression screening as part of the overall eligibility assessment and recruitment process.

Research staff outreach and referral: Research staff visited community agencies and attended community events, such as resource fairs, at which service agencies advertise their programs. Staff provided interested women with information about the intervention project, screened for inclusion criteria, and referred mothers to the project coordinator for enrollment.

Online maternal self-referral: The project maintained a self-referral mechanism through its website, which provided the following: (1) access to a brief video describing the intervention programs; (2) information about the project team; (3) depression screening; and (4) a form for providing contact information to research staff. To promote awareness of the online self-referral mechanism, the research team posted information on local community agency websites, social media platforms, and in print material available at local community agencies. We did not use any paid advertising mechanism.

### 2.3. Measures

To assess maternal progression from referral through successful recruitment into the study intervention, the following variables were documented by date of occurrence or disposition within the project database: referred, screened for eligibility, and eligible after screening. The PHQ-2 was administered online to screen for depression with the established criteria of a score of 3 or higher defined as a positive depression screen. The PHQ-2 is an efficient and well-established measure with strong psychometric characteristics for identifying individuals with depression [37]. At pre-intervention assessment, participants completed a demographic questionnaire to facilitate characterization of the sample with regard to mother’s age, ethnicity, race, educational level, income, significant relationship status, and number of children in the home. We also obtained child age in months and child sex.

Additional participant intrapersonal risk characteristics were also assessed at pre-intervention. The Patient Health Question-9 (PHQ-9) was administered to assess depression severity. Endorsement of the PHQ-9 item, “Thoughts that you would be better off dead or of hurting yourself”, was viewed as an indicator of self-harm thoughts [38]. The PHQ-9 possesses strong psychometric properties for assessing depression severity; a score at or above 20 is suggestive of severe depression [39]. Participants were also administered the Knowledge of Infant Social communication Development and Competency Promotion, which has demonstrated high internal consistency and sensitivity to intervention change [40].

### 2.4. Analysis

Using data collected between 21 September 2018 and 20 September 2019 on 203 referred mothers, we viewed the following five progression points into intervention: number of mothers referred from each of the three approaches, the number of mothers screened for eligibility, the number of mothers found to be eligible in screening, the number of mothers who consented to participate in the clinical trial, and the number of mothers who initiated the intervention. For number referred, we do not know the number of mothers within each approach, who could possibly have been referred in order to calculate a relative rate of referral within each approach. We will, therefore, report each approach’s contribution of referred mothers to the overall recruited sample and calculate a multiplicative index to reflect any referral number differences between approaches (e.g., one approach contributed 2.7 times the number of referred mothers from other approaches across the same time period). The examination of other successive efficiency progression points is dependent upon the sample sizes resulting from each referral approach. Utilizing G*Power [41] with an overall *n* of 203, assuming equal numbers of mothers within each referral condition, with *p* < 0.05 two-tailed, we calculated that we would have less than 80% power (i.e., 71%) to detect moderately small effect sizes (d = 0.40), between the three conditions. If sample sizes diverge between the referral approach groups, parametric power would be reduced even further. Hence, to reduce the power burden, we took a conservative approach and limited analysis a priori to a two-group comparison, comparing maternal self-referral and agency referral groups. The a-priori group comparison was based on the fact that the two referral conditions selected are the most salient for referral into intervention research. The Chi Square test was used for the first research question and the Mann–Whitney tests were used for the second research question, given categorical versus ordinal variables, respectively. Working with an equal n two-group design (*n* = approximately 136), using wmwpow [42] at 80% power, small effect size (d = 0.40), *p* < 0.05 two-tail, and assuming a normal distribution for the two groups, we estimated power = 0.80, an acceptable criterion. If groups sizes differed, however, power was reduced and we focused on reporting effect size estimates, viewing d = 0.30 or higher as potentially meaningful for subsequent examination. 

## 3. Results

**Figure 1:**
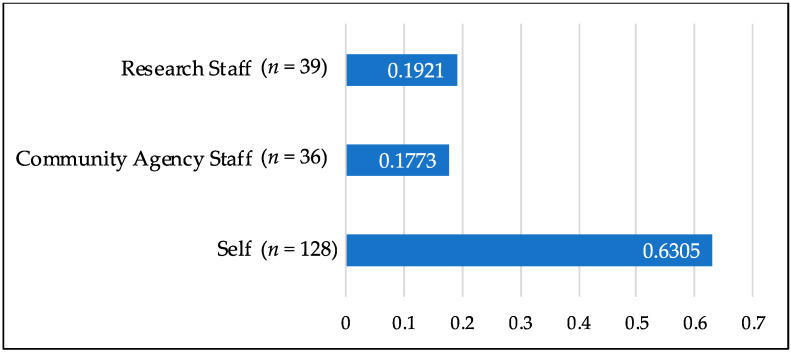
Percentage of each referral group contribution to the total referred sample.

With regard to the first research question on referral success, the number of self-referred mothers far surpassed that of mothers referred from traditional agency and research referral gatekeepers, with more than 3.5 times more referrals generated from the self-referral group as compared to referrals from community agency staff or research staff referral groups (see Figure 1).

The large difference in the number of cases generated through self-referral, as compared to agency and research staff, had significant impact on the number of cases to be examined at each successive point. Hence, we moved to our most conservative test to reduce burden on power, as described earlier. We conducted four Chi Square tests of between-group examinations and restricted comparisons to the self-referral and agency-referral groups. Table 2 presents the number of mothers within each referral group at each successive point.

**Figure 2:**
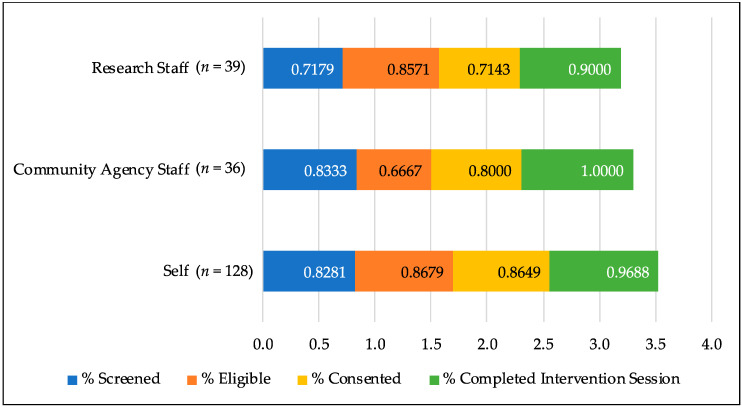
Success of referral to intervention engagement by referral strategy.

Overall, agency-referred and self-referred mothers moved at similar percentage rates through four of the five successive points. The number of women who were eligible to participate based on the screening, however, was significantly higher for the self-referred group as compared to the agency-referred group. This difference reflected a small effect (Chi Square = 6.52, *p* = 0.01, d = 0.45). Figure 2 presents an overall view of the proportion of mothers moving through each point of the recruitment process by referral strategy.

Our second question focused on intrapersonal risks experienced by referred mothers. As displayed in the demographics shown in Table 1 above, we achieved our intended sample of mothers, who were primarily of non-dominant culture (Black race) and socioeconomically disadvantaged who, as established in the literature review, experience extreme inequity in accessing depression-focused intervention. We, therefore, created a cumulative intrapersonal risk index to view the level of other risk variables over race and poverty among mothers in our recruited sample (See Table 3). Each of the five risk variables was dichotomized based on the criterion specified in the table, with 1 representing presence of the characteristic. These variables were summed to produce a risk index range of 0–5.

**Figure 3:**
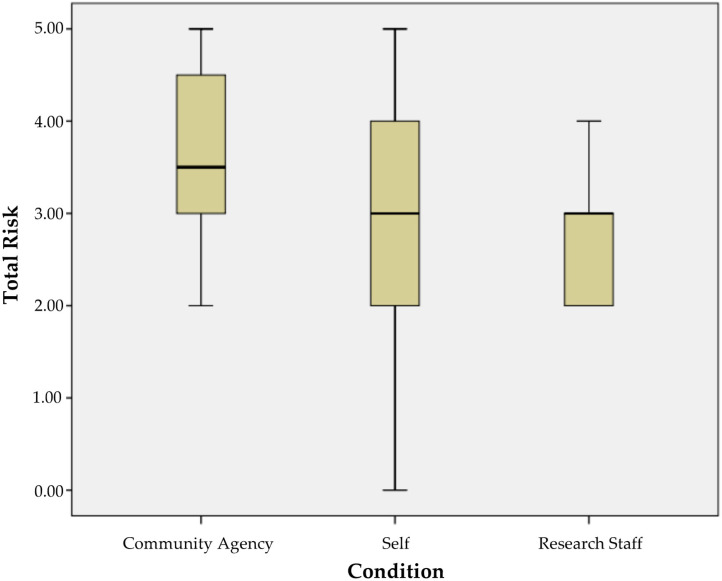
Boxplot of risk for agency, self-referral, and research staff approaches.

Figure 3 presents a plot of the risk index for mothers recruited using each referral strategy. Descriptively, mothers referred by research staff had the lowest and most restricted range of risk. The self-referred mothers had the largest range of risk. Agency-referred mothers demonstrated a slightly higher 75th percentile value (approximately 4.5/5 risks) than those of self-referred mothers (approximately 4/5 risks). For staff-referred, the 75th percentile risk level was approximately the same as the median risk value for agency-referred mothers. We followed the conservative approach to examination and conducted a Mann–Whitney between-group comparison of agency-referred and self-referred mothers. This examination resulted in a small effect size difference (U = 268; *p* = 0.08, d = 0.39), with agency-referred mothers reporting slightly higher levels of risk.

## 4. Discussion

### 4.1. Summary

The gatekeeper referral systems that relied on agency- and research-staff referrals were less successful compared to mother self-referral. They resulted in substantially fewer initial referrals and experienced losses of potential participants at rates equivalent to or sometimes greater than that of the self-referral system at each stage of the process up through initiation of the intervention. A substantial proportion of mothers across referral groups consented to participation and initiated the intervention. As planned, the final sample across referral groups reflected the population from which they were recruited: non-dominant culture, experiencing severe socioeconomic disadvantage, not having a significant other, and having limited knowledge of infant social-emotional development.

In our preliminary examination of risk factors experienced by mothers, mothers in the self-referred group had the greatest range of risk levels. Though this almost certainly reflects the relatively larger size of the group relative to those referred through other mechanisms, it nonetheless suggests that this approach has potential to result in a somewhat diverse group of participants with regard to these factors. It is important to note that mothers referred by agency staff evidenced a somewhat higher level of risk factors. Though the effect size was small, and the small sample size renders the finding preliminary, it is consistent with prior evidence that individuals with greater resources are more likely to bypass gatekeeper systems to access mental health services directly [28]. We think these findings suggest the potential importance of continuing to try to engage community agency gatekeepers in the referral process. It seems plausible that busy agency staff may be reserving their efforts for women whom they see as most vulnerable. It is also possible that though self-referral is excellent with regard to creating a smooth path to entry for most women (including those who are depressed, of non-dominant culture, and socioeconomically disadvantaged), it is not yet clear the threshold of vulnerability at which entry may require too much initiative for mothers.

### 4.2. Contributions

Although online recruitment is common in mobile health interventions, there is limited research on recruitment [32]. To our knowledge, this is a first systematic examination of referral processes as they relate to recruitment into intervention for depressed mothers. The existing research on online recruitment into parenting interventions for mothers of newborns has focused nearly exclusively on paid advertising such as through ads on parenting sites and via Facebook [33]. In contrast, our study provides an examination of online recruitment that did not require any paid advertising—a potentially important consideration for cost containment. Although published studies have been reported on the use of non-paid advertising for recruiting into mobile health interventions focused in areas such as smoking cessation [34], they have tended to yield less diverse samples, reflecting primarily dominant culture groups [34]. The present study is unique in that it focuses on a target sample of postpartum women with depression, who are lacking in existing study samples, namely women who identify as non-dominant culture and who are socioeconomically disadvantaged. 

### 4.3. Limitations and Implications for Future Research

The current study has important limitations to note, which reflect that this was a secondary analysis of existing data to address an important research question that was distinct from those of the original parent study. First, relative to race, ethnicity, and income demographics of the sample, our clinical trial is being conducted within an area of concentrated poverty in the urban center of a large southern city, where the population is predominantly Black. Moreover, because we sought to refer and recruit within agencies serving low income individuals within one of the most income disparate cities in the U.S. with a long history of structural and systemic racism [43], the majority of our sample is severely socioeconomically disadvantaged. As such, we cannot adequately examine race and income sample differences by referral group. The current results may not be generalizable to other referral and recruitment efforts taking place within target populations that contain greater diversity in race and income. Of course, it seems unlikely that an approach that worked this successfully in a highly stressed and low resourced population would not be feasible in other samples.

A second limitation is the lack of clinical diagnostic measures. As the aims of the larger study did not necessitate diagnostic interviews, depression is defined here by a well-established questionnaire measure, which, though it has strong concordance with clinically derived diagnoses, is not equivalent and does not provide indices of cooccurring disorders. As such, we do not know how many of the participants met diagnostic criteria for current mood disorders or whether the presence of depressive or comorbid diagnoses influence the success of one or more of the referral processes. Nonetheless, the current results suggest that women experiencing depressive symptoms at levels likely to indicate disorder were enrolled successfully.

Another limitation is the relatively small sample. Clearly, future research on larger samples within more regionally, socioeconomically, and racially diverse target populations is needed to determine generalizability of the current findings and, in particular, to provide stronger data regarding the extent to which diverse recruitment strategies yield equivalent samples. However, based on the current results, we note evidence in support of the possibility that mothers who are Black and socioeconomically disadvantaged can and do self-seek services beyond typical gatekeeper systems to address depression. Studies are needed within community settings, outside of the clinical intervention trial structure, to determine if self-referral into intervention will result in greater racial and ethnic equity in accessing needed community services to reduce depression and strengthen parenting. It is also important to note that our examination of referral progression toward intervention access took place within the framework of a clinical intervention trial. As such, the outcomes may not generalize to processes of community agency and self-referral into community-based clinical intervention services, where there may be less support for moving mothers toward intervention access.

Expanding beyond the current study, other important future research directions to consider include the following: (1) examination of other factors that might influence referral such as insurance status, number of children in the home, or feelings about online interventions; (2) examination of cost effectiveness of various referral approaches; (3) documentation of how self-referring mothers access recruitment websites to self-refer (for example, whether by content searches, service searches, in response to friend suggestions in social communications, or responding to print or electronic links from trusted service providers), and (4) studies that extend beyond the relationship between referral approach and intervention access to include exploration of the relationship between referral approach and study retention, especially with regard to intervention dosage.

## 5. Conclusions

Results showed that mothers self-referred at more than 3.5 times the rate of referral by community agency staff and research staff. The resultant sample across referral groups reflected mothers of Black race experiencing severe socioeconomic disadvantage. Compared to traditional referral gatekeeper groups, self-referred mothers were equally successful with regard to recruitment into the study intervention.

## Figures and Tables

**Table 1:** Demographic characteristics of the sample recruited into the study.

Variable	Value
Maternal age in years, mean (SD); range	28.12 (5.85); 19.00 to 44.00
Child age in months, mean (SD); range	5.16 (2.82); 2.00 to 12.00
Number of children in the home, mean (SD); range	2.67 (1.45); 1.00 to 6.00
Maternal race (Black) % (*n*)	95.35% (82)
Maternal ethnicity (Latinx), % (*n*)	3.49% (3)
Maternal Education (<college degree), % (*n*)	84.88% (73)
Maternal income, %(*n*) </=138% Federal Poverty Guideline	85.00% (68)

**Table 2:** Number of mothers by referral group meeting each progression point.

Variable	Agency Referral	Research Staff Referral	Mother Self-Referral	Total Referrals
Number referred	36	39	128	203
Number/% screened for eligibility	30	28	106	164
	83.33%	71.79%	82.81%	80.79%
Number/% eligible based on screening	20	24	92	136
	(66.67%)	(85.71%)	(86.79%)	(82.93%)
Number/% who consented	12	10	64	86
	(60%)	(41.67%)	(69.56%)	(63.23%)
Number/% who completed initial intervention session	12	9	62	83
	(100%)	(90%)	(96.88%)	(96.51%)

**Table 3:** Intrapersonal risk characteristics by referral approach group.

Variable	Agency Referral	Research Staff Referral	Mother Self-Referral
* *N*/% Less than college degree	12	10	51
	100%	100%	79.69%
*N*/% Severe symptom range	5	2	21
	41.67%	20%	32.81%
* *N*/%Thoughts of self-harm	6	0	11
	50%	0%	17.19%
No significant other	9	7	46
	75%	70%	71.88%
*N*/% < 60% Parent knowledge of infant SE development and promotion	11	10	53
	91.67%	100%	82.81%

* Significance level <0.05.
